# Review of a three-year study on the dental care of onco-hematological pediatric patients

**DOI:** 10.6061/clinics/2017/e721

**Published:** 2018-11-23

**Authors:** Alexandre Viana Frascino, Marcelo Fava, Louise Cominato, Vicente Odone-Filho

**Affiliations:** Instituto da Crianca (ICr), Hospital das Clinicas HCFMUSP, Faculdade de Medicina, Universidade de Sao Paulo, Sao Paulo, SP, BR

**Keywords:** Pediatric Dentistry, Dental Care, Ambulatory Care, Neoplasms

## Abstract

The aim of this study was to provide an updated review of dental procedures undertaken at the dental unit of the Onco-hematology service of the Instituto da Criança at the Hospital das Clínicas, Faculdade de Medicina, Universidade de São Paulo (ICr/HC-FMUSP).

We retrospectively reviewed 565 of 1902 medical and dental records of patients diagnosed with onco-hematological diseases who were seen in a 3-year study (January 2015 to December 2017). We assessed data regarding population characteristics, onco-hematological diagnosis and dental procedures performed.

Of the selected medical records, preventive dentistry was the most common procedure undertaken in this population, followed by oral maxillofacial surgeries, restorative dentistry and oral mucositis treatment. The most prevalent malignant diagnosis was acute lymphocytic leukemia, and the most prevalent nonmalignant diagnosis was sickle-cell anemia.

Preventive dental procedures represent most of the dental procedures undertaken in hospitalized onco-hematological pediatric patients.

## INTRODUCTION

The survival rate of pediatric onco-hematologic patients has increased in the past few years due to a combination of clinical approach, surgery, radiotherapy, chemotherapy and stem cell hematopoietic transplantation (HSCT) techniques. However, short- and long-term complications have been reported 1,2.

Oral problems resulting from cancer therapy, such as oral mucositis, which negatively affects body weight gain in children undergoing HSCT 3, increase the risk of local and systemic infections 4, length of hospital stay 5-7 and treatment cost 8 and impair oral health-related quality of life (OHRQoL) 9,10. Bezinelli et al. (2016) reported an improvement in OHRQoL after proper treatment for oral mucositis in pediatric patients undergoing HSCT 11. The multidisciplinary approach in pediatric oncology care results in increases in quality of life 12,13.

The dental unit of the Onco-hematology service of the Instituto da Criança at the Hospital das Clínicas, Faculdade de Medicina, Universidade de São Paulo (ICr/HC-FMUSP) was established in 2007 with the aim of offering proper dental care to pediatric patients undergoing onco-hematological therapies. In the first year of its inauguration, the most common dental procedures among the study population were restorative dentistry, preventive procedures and the removal of infectious foci 14.

The aim of this paper was to provide an updated review of dental procedures undertaken at the dental unit of the Onco-hematology service of ICr/HC-FMUSP, including population characteristics, onco-hematological diagnosis and the distribution of dental procedures performed annually.

## MATERIALS AND METHODS

**Figure 1:**
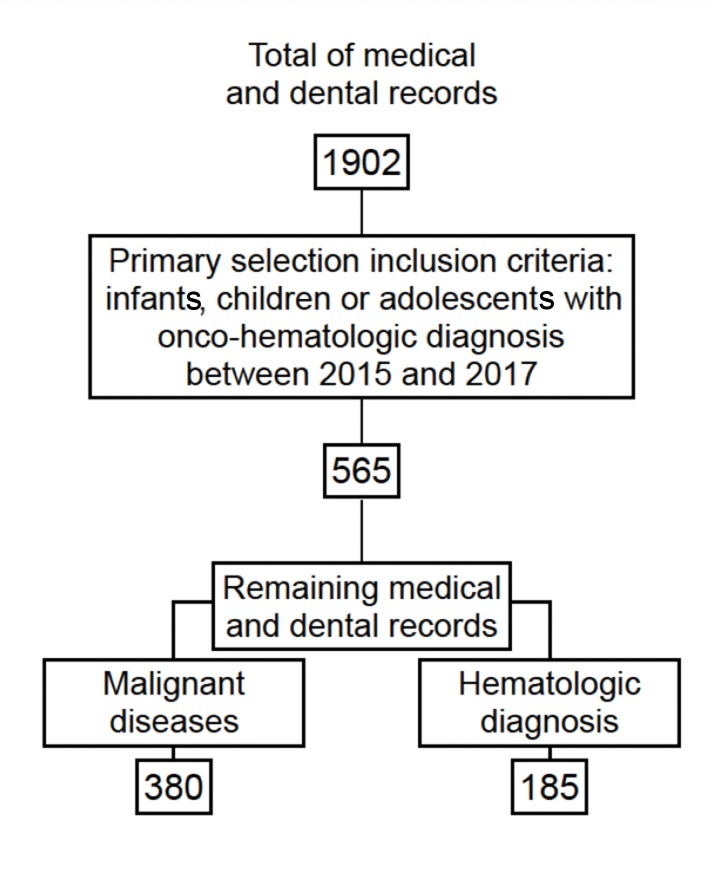
Inclusion/exclusion criteria flowchart for the selection of medical records and distribution of medical diagnosis.

The medical and dental records of 1902 patients seen at the dental unit of the onco-hematology service of ICr/HC-FMUSP during the 3-year study were reviewed. Only 565 patients achieved the inclusion criteria: infants, children or adolescents (0 to 18 years old) who were diagnosed with onco-hematological disease between January 2015 and December 2017. A total of 1307 medical records were excluded due to erroneous information, unconfirmed onco-hematological diagnosis or death (Figure 1).

We collected data concerning gender, age and primary cancer diagnosis and categorized dental procedures performed in the last three years as either preventive or therapeutic.

### Ethics

All the primary caretakers gave their signed approval to provide medical records for this study. No biological material was collected, and the study was conducted in accordance with the ethical standards of the responsible committee on human subjects. Individual information remained confidential, and all patient identifiers, such as names, addresses and personal characteristics, were thoroughly preserved in this investigation.

This research was approved by the Faculdade de Medicina da Universidade de São Paulo ethics committee in May 2015 (protocol number: 1.073.602).

## RESULTS

**Figure 2:**
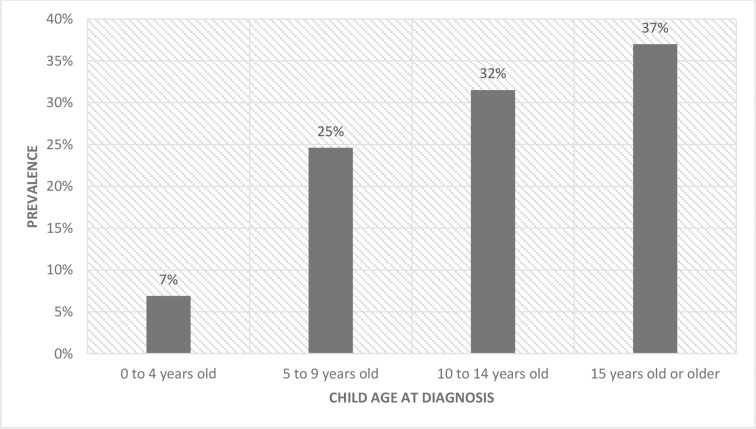
Age distribution of the study population. The prevalence of age groups in children most commonly affected by each type of cancer in the study population.

Of the 565 medical records selected for thorough review, 380 (66.73%) were diagnosed as malignant, and 185 (32.74%) were diagnosed as hematological. In total, 53% of the population was male, and 47% was female. The mean age at diagnosis was 11.5 years (SD: 4.22; MIN: 1; MAX: 18), and the age distribution is shown in Figure 2.

**Figure 3:**
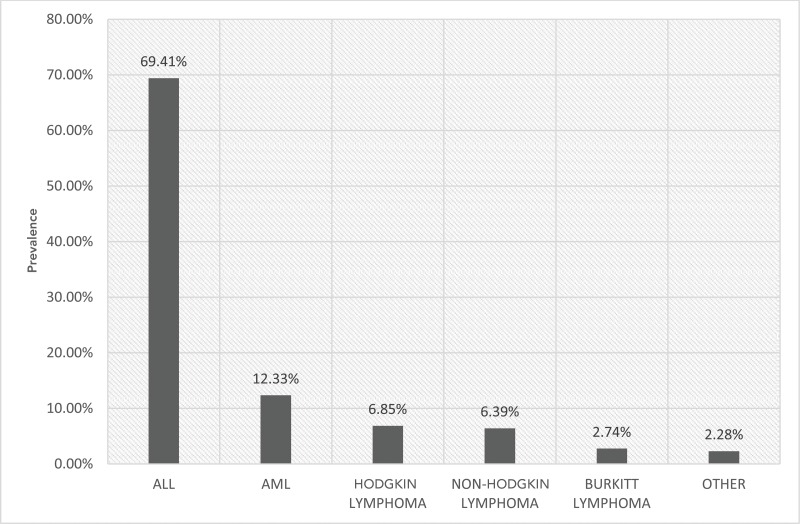
Distribution of malignant diagnoses. ALL: acute lymphocytic leukemia; AML acute myeloblastic leukemia. “Other” refers to chronic lymphocytic leukemia, B-cell lymphoma and lymphoblastic lymphoma.

The most prevalent malignant diagnosis was acute lymphocytic leukemia (ALL) (69.41%), followed by acute myeloblastic leukemia (AML) (12.33%), Hodgkin lymphoma (6.85%), non-Hodgkin lymphoma (6.39%) and Burkitt lymphoma (2.74%). Other malignant diagnoses comprised 2.28% altogether. Figure 3 provides the detailed distribution of malignant diagnoses.

**Figure 4:**
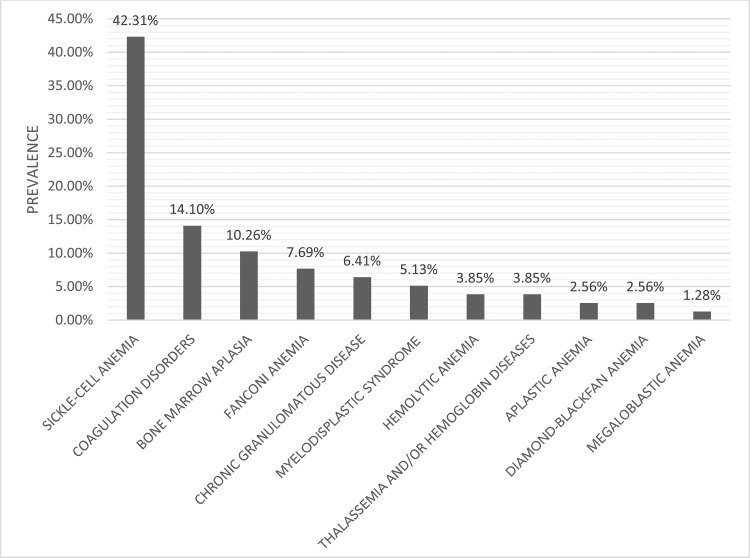
Distribution of nonmalignant diagnoses. Sickle-cell anemia was the most prevalent diagnosis among the nonmalignant diseases.

Of the nonmalignant diseases, the most prevalent diagnosis was anemia (70.51%). Of these, sickle-cell anemia comprised 42.31% of the cases. Figure 4 provides the detailed distribution of nonmalignant hematological diagnoses.

Between 2015 and 2017, 1411 dental procedures were performed at the dental unit of the Onco-hematology service of the Instituto da Criança at the Hospital das Clínicas, Faculdade de Medicina, Universidade de São Paulo.

**Figure 5:**
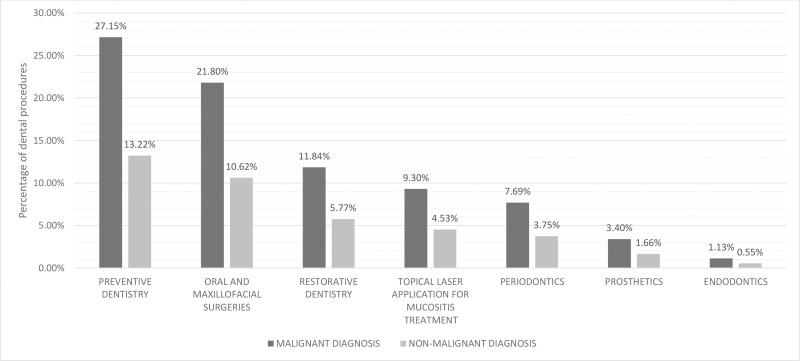
Dental procedures performed in malignant and nonmalignant patients (%). Distribution of dental procedures showing the proportions of malignant and nonmalignant groups.

**Figure 6:**
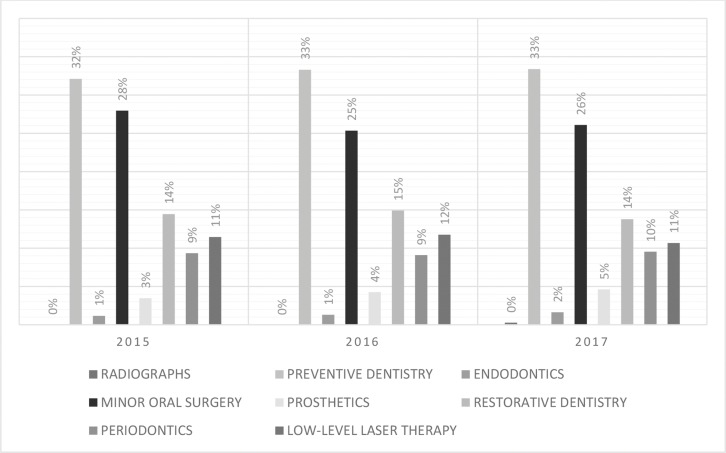
Annual distribution of dental procedures.

The distribution of dental procedures shows the proportions of both malignant and nonmalignant groups. In total, preventive dental care was the most commonly performed dental procedure (n=465; 33%). Oral and maxillofacial surgeries comprised 26% of the dental procedures (n=373, 26%). Restorative dentistry comprised 14% (n=203), and topical laser application for oral mucositis comprised 11% (n=159). In total, gum treatments (periodontics) and root canal filing (endodontics) encompassed 15% of the dental procedures. Figures 5 and 6 show the distribution of dental procedures among the studied groups.

## DISCUSSION

The survivorship and free-event lifetime of childhood onco-hematological malignancies have increased due to the combination of multiple therapies that includes more effective drugs for long-term clinical follow-up, less invasive and more conservative surgeries, multiple agent chemotherapy, radiotherapy and hematopoietic stem cell transplantation 15,16. Several oral complications have been associated with antineoplastic therapies that cause pain and discomfort, require parenteral narcotic therapy and extended hospitalizations, increase costs, and interfere with the course of treatment and prognosis of the neoplasm 13,17-22.

Age and gender distributions found in the present study were consistent with those reported in previous publications, indicating that in Brazil, leukemia is more prevalent in boys aged 1 to 4 years old (31.6%), lymphoma is dominant in males between 15 and 18 years old (35.6%), and tumors of the central nervous system have a similar male:female ratio (26%) in patients under 14 years old 17.

Preventive dental procedures, such as topical fluoride application and professional oral hygiene, were most common (32.94%). These preventive procedures reduced the frequency of restorative treatment and the prevalence of infectious foci 14 and were consistent with the American Academy of Pediatric Dentistry (AAPD) dental care guidelines for pediatric cancer patients. Oral hygiene orientation, topical fluoride application and patient/parent education helped to minimize oral discomfort during treatment 23.

Oral mucositis is a debilitating dose-related complication that commonly affects hospitalized patients undergoing cancer therapy and is often addressed by a dental unit team. Oral mucositis manifests as a burning sensation in the oral mucosa and can lead to the formation of edema, erythema and ulcers. Topical laser application is a well-established and noninvasive approach that effectively controls oral mucositis 24-26. In our study, 11% of the dental protocols consisted of topical laser applications. This result emphasizes the importance of multidisciplinary care in reducing pain and discomfort in hospitalized patients.

Another important aspect of preventive multidisciplinary care in hospitalized patients is related to the unavailability to work during chemotherapy or radiotherapy 18,27,28. To overcome this issue, the dental unit of the Onco-hematology service of ICr/HC-FMUSP was established in 2007 to provide specialized dental care to hospitalized pediatric patients and to fully coordinate oncologists and pediatricians on matters concerning diagnosis, prognosis, treatment and patient discharge.

Long-term follow-up studies with information regarding the oral conditions of pediatric onco-hematological patients are needed. These studies can enhance dental care in this population and therefore improve quality of life. Additionally, further studies are necessary to establish specific dental protocols suitable for short- and long-term cancer therapies and improve not only survivorship but also quality of life.

Based on the present study, it can be concluded that preventive dental procedures were performed mostly in onco-hematological pediatric patients, followed by minor oral surgeries and restorative dentistry. Longitudinal and cohort studies are needed.

## AUTHOR CONTRIBUTIONS

Frascino AV was the main author and researcher. Fava M was responsible for the data review and organization. Cominato L was responsible for the medical records review. Odone-Filho V was the research advisor.
